# O-Hexadecyl-Dextran Entrapped Berberine Nanoparticles Abrogate High Glucose Stress Induced Apoptosis in Primary Rat Hepatocytes

**DOI:** 10.1371/journal.pone.0089124

**Published:** 2014-02-20

**Authors:** Radhika Kapoor, Shruti Singh, Madhulika Tripathi, Priyanka Bhatnagar, Poonam Kakkar, Kailash Chand Gupta

**Affiliations:** 1 Food, Drug and Chemical Toxicology Division, CSIR- Indian Institute of Toxicology Research (CSIR-IITR), Lucknow, Uttar Pradesh, India; 2 CSIR- Institute of Genomics and Integrative Biology, Delhi University Campus, Delhi, India; Central Michigan University School of Medicine, United States of America

## Abstract

Nanotized phytochemicals are being explored by researchers for promoting their uptake and effectiveness at lower concentrations. In this study, O-hexadecyl-dextran entrapped berberine chloride nanoparticles (BC-HDD NPs) were prepared, and evaluated for their cytoprotective efficacy in high glucose stressed primary hepatocytes and the results obtained compared with bulk berberine chloride (BBR) treatment. The nanotized formulation treated primary hepatocytes that were exposed to high glucose (40 mM), showed increased viability compared to the bulk BBR treated cells. BC-HDD NPs reduced the ROS generation by ∼3.5 fold during co-treatment, prevented GSH depletion by ∼1.6 fold, reduced NO formation by ∼5 fold and significantly prevented decline in SOD activity in stressed cells. Lipid peroxidation was also prevented by ∼1.9 fold in the presence of these NPs confirming the antioxidant capacity of the formulation. High glucose stress increased Bax/Bcl2 ratio followed by mitochondrial depolarization and activation of caspase-9/−3 confirming involvement of mitochondrial pathway of apoptosis in the exposed cells. Co- and post-treatment of BC-HDD NPs prevented depolarization of mitochondrial membrane, reduced Bax/Bcl2 ratio and prevented externalization of phosphatidyl-serine confirming their anti-apoptotic capacity in those cells. Sub-G1 phase apparent in high glucose stressed cells was not seen in BC-HDD NPs treated cells. The present study reveals that BC-HDD NPs at ∼20 fold lower concentration are as effective as BBR in preventing high glucose induced oxidative stress, mitochondrial depolarization and downstream events of apoptotic cell death.

## Introduction

Diabetes mellitus is a chronic metabolic disorder caused by relative deficiency of insulin secretion and is characterized by high circulating glucose [1]. By 21^st^ century, it has attained the status of the most challenging and unresolved problem. Around the globe, ∼230 million people have been affected by the disorder and the number is estimated to reach ∼366 million by 2030 [2]. Various pathways involved in glucose metabolism such as polyol pathway, sorbitol pathway, Advance Glycation End product (AGE) pathway and hexosamine pathway are considered to be responsible for the generation of reactive oxygen species (ROS) during high glucose stress. Due to continuous high circulating glucose during diabetes, natural antioxidant defence system is compromised leading to generation of oxidative stress. Excessive generation of ROS has been shown to be responsible for metabolic abnormalities and chronic complications [3]. Numerous studies have reported co-relation between oxidative stress onset, progression of diabetes and its associated complications [4]. Hence, an effective and beneficial strategy for management of diabetes can be through the use of antioxidants, which can effectively ameliorate the ROS or stress generated due to hyperglycemia. Treatment with insulin or chemically derived drugs has limitations as they exhibit various complications like insulin resistance, anorexia nervosa, brain atrophy and fatty liver [5]. Therefore, there is a need to find a pharmacologically efficacious molecule which is cost-effective and can prevent oxidative stress mediated damage to cells during hyperglycemia.

In ayurvedic and chinese medicine, medicinal plants containing berberine chloride, an alkaloid, have been used since long time. It is present in *Hydrastis canadensis* (goldenseal), *Coptis chinensis* (Coptis or golden thread), *Berberis aquifolium* (Oregon grape), *Berberis vulgaris* (barberry), and *Berberis aristata* (tree turmeric). The alkaloid is obtained from roots, rhizomes and stem bark of these plants. The plant extracts and decoctions have been known to possess antimicrobial activity against several types of infection viz., bacteria, virus, fungi, helminths, intestinal parasite infections and ocular trachoma infections [6]. Apart from various beneficial effects of berberine chloride; it suffers from some biopharmaceutical limitations due to its poor aqueous solubility and foul taste [7].

Nano particles mediated drug delivery systems containing phytochemicals from traditional medicines having advanced pharmacokinetic and pharmacodynamic properties can be explored as newer therapeutic strategies. These nano-vehicles offer unique features, such as enhancing the solubility and bioavailability of the drug, prolonging the circulation time, preferential accumulation at the target organ and reducing systemic side effects [8], [9]. Hence, solubilising berberine chloride into a readily soluble form, entrapping it into a system that delivers the molecule fast into the organ system can be a better option for exploring and using the medicinal properties of berberine against hyperglycemia.

Over the past years, many polymers have been examined for drug delivery applications including natural polymer hydrogels (cellulose, albumin, collagen, dextran, chitosan, etc.) and synthetic polymers (polyesters, polyanhydride, poly(alkyl cyanoacrylate), etc.) [10]–[13]. Among various polymers, dextran has been studied extensively. Dextran, a polysaccharide consisting of a linear α-1,6-glycosidic linkage with some degree of branching via a 1,3-linkage, has extensively been used in food and medical fields [14]–[16]. It has been employed in a range of biomedical applications because of its excellent aqueous solubility, biocompatibility, and non fouling properties [17], [18]. It has been suggested as a potent drug carrier owing to several advantages over the others such as well defined structure, high stability of glycosidic bonds, low pharmacological activity, low toxicity and protection of conjugated drugs from biodegradation [19]. Also, dextran nanoparticles manage to escape from the uptake by mononuclear phagocytic system (MPS) macrophage and thus larger plasma persistence as well as prolonged therapeutic effects. In addition, presence of numerous reactive hydroxyl groups in dextran allows its derivatization thereby, providing wide range of properties [20].

Therefore, in the present study, we selected dextran, which was first chemically modified by the covalent attachment of aliphatic hydrocarbon chain through the formation of ether linkages. Thereafter, dextran derivative was used as the polymeric material for encapsulating berberine and further examined for its anti-diabetic responses in rat hepatocytes against the bulk berberine. Further, the study was aimed to explore the possibility of using HDD entrapped berberine NPs for their cyto-protective efficacy (reduced oxidative stress) in high glucose stressed primary rat hepatocytes. We showed that O-hexadecyl-dextran entrapped berberine chloride nanoparticles (BC-HDD NPs) have improved cytoprotective effect on high glucose stressed primary hepatocytes by altering many critical control points of mitochondria-mediated apoptosis.

## Materials and Methods

### Animals

Animal handling in all experimental procedures was approved by the Institutional Animal Ethics Committee of CSIR-Indian Institute of Toxicology Research (ITRC/IAEC/20/2010). Male Wistar rats weighing 200±20 g from Indian Institute of Toxicology Research (IITR) animal colony were used for the isolation of primary hepatocytes. Rats were housed in an air conditioned room at 25±2°C temperature with 60–70% humidity and a controlled 12 h light/dark cycle. Rats were fed on standard pellet diet (Ashirwad Pellet Diet, Mumbai, India) and water *ad libitum*.

### Chemicals

RPMI-1640, Berberine chloride, FBS (Foetal bovine serum), DCFH-DA (2′,7′,-Dichlorohydrofluorescein Diacetate), 5,5′,6,6′-tetrachloro 1,1′,3,3′-tetraethylbenzimidazol carbocyanine iodide (JC-1), Rhodamine 123, Hoechst 33258 fluorescent probes, MTT (3-(4,5-Dimethylthiazol-2-yl)-2,5-diphenyltetrazolium bromide), Phosphate Buffered Saline (PBS) were purchased from Sigma-Aldrich (St. Louis, MO, USA). CellTracker™ Green CMF-DA (5′-chloromethylfluorescein diacetate) fluorescent dye was procured from Molecular probes (Eugene, Oregon, USA). Primary antibodies against anti- Bcl-2, Bax, Caspase-3, Caspase-9 and horse radish peroxidase-conjugated secondary antibody (2° Ab) were procured from Santa Cruz Biotechnology, Inc. whereas anti- β-actin was purchased from Sigma-Aldrich (St. Louis, MO, USA). All the solutions were prepared in ultrapure deionised water (Direct Q5, Millipore, Bangalore, India). Unless mentioned otherwise, all the chemicals were purchased from Sigma Chemicals Co. (St. Louis, MO, USA).

### Preparation and Characterization of Berberine Nanoparticles

#### Preparation of O-hexadecyl-dextran (HDD)

Dextran (1 g) and sodium hydroxide (400 mg) were dissolved in double distilled (dd) water (10 ml). After complete dissolution, 1-bromohexadecane (550 µl, for 30% substitution) in tetrahydrofuran (THF, 10 ml) was added and the reaction mixture was stirred overnight at 80°C. Solvent was removed on a rotary evaporator; the remaining aqueous solution was diluted with water (10 ml) and extracted with dichloromethane (2×10 ml) to remove unreacted 1-bromohexadecane. The aqueous phase was collected, subjected to dialysis for 24 h and subsequently lyophilized to obtain the O-hexadecyl dextran (HDD) in ∼82% yield, which was characterized by ^1^H-NMR (D_2_O) δ:1.17–2.0 (m, -CH_2_-), 3.25–3.9 (m, -OCH-, -OCH_2_-), 5.17–5.23 (m, -CH_2_-).

#### Encapsulation of berberine chloride in HDD (BC-HDD)

Berberine chloride encapsulated HDD NPs were prepared by adding HDD (120 mg) to a solution of berberine chloride (20 mg), dissolved in dd water (4 ml), and the mixture was stirred for 6 h at 25±2°C. The reaction mixture was then dialysed for 2 h against water and freeze-dried to obtain free-flowing BC-HDD NPs.

#### Characterization of BC-HDD NPs-Size and zeta potential measurements

Particle size and distribution along with zeta potential measurements of the BC-HDD NPs were carried out by Dynamic Light Scattering (DLS) using a Zetasizer Nano-ZS (Malvern Instruments, U.K.). A measured amount of the BC-HDD NPs were suspended in water (1 mg/ml) and the mean particle size and charge were measured employing the following settings on the instrument; refractive index of water, 1.33; viscosity for water, 0.89 cP. All measurements were carried out at 25±2°C. Zeta potential measurements were carried out in triplicates in automatic mode and the values presented as the average value of 30 runs. The Smoluchowski approximation was used to calculate zeta potential from the electrophoretic mobility.

#### Transmission electron microscopy

The surface morphology and size of the BC-HDD NPs were analyzed using Transmission Electron Microscopy (TEM). Briefly, aqueous solution of freeze dried nanoparticles was prepared at the concentration of 1 mg/ml. A drop of this solution was placed on a TEM grid surface followed by the addition of a drop of 1% uranyl acetate onto the grid surface. After 1 min of incubation, excess fluid was removed and the grid surface was air dried at 25±2°C before loading onto the microscope. Then the NPs were observed under an electron microscope (FEI Tecnai G2 sprit Twin Transmission Electron Microscope equipped with CCD Camera, Netherlands) at 80 kV.

#### Determination of encapsulation efficiency

The encapsulation efficiency of berberine in BC-HDD NPs was determined by a spectrophotometric method. In brief, 10 mg of BC-HDD NPs were suspended in acetonitrile:water (1∶1, v/v, 1 ml) and the suspension was vortexed for 10 min. The pellet was collected after a brief centrifugation at 10,000 g for 30 min and measured the absorbance of the supernatant at 263 nm. The amount of drug (mg) entrapped was calculated from the standard curve drawn between the varied amount of drug (mg) and absorbance (O.D.). The following equation was used to determine the encapsulation efficiency (EE). All the measurements were conducted in triplicate. Encapsulation efficiency (%) = (weight of BC in BC-HDD NPs/weight of drug used)×100.

#### In vitro release of BC from BC-HDD NPs

To evaluate the release pattern of BC from the BC-HDD NPs, 10 mg of nanoparticles were suspended in phosphate buffered saline (1×PBS, 1 ml, pH 7.4) and poured in the dialysis tube, which was suspended in 1×PBS (20 ml) in a glass bottle. The solution was slowly stirred (∼150 rpm) at 37°C and at pre-determined intervals of time; the sample was collected (ca. 500 µL) from the glass bottle and measured its absorbance at 263 nm spectrophotometrically. Same amount of fresh buffer was added to the glass container and the release study was continued. Quantity of the released drug was then calculated using a previously drawn standard curve of the pure drug in phosphate buffer.

### Cell Culture

Primary hepatocytes were isolated from liver of overnight fasted rat according to the two step collagenase perfusion method [21]. Cell viability was checked by trypan blue dye exclusion test within 1 h of cell isolation. Hepatocytes were maintained in RPMI-1640 media supplemented with heat inactivated 10% fetal bovine serum and 1% of 10,000 units Penicillin, 10 mg Streptomycin, 25 µg Amphotericin B, 1 mM sodium pyruvate, 2 mM glutamine under an atmosphere of 5% CO_2_–95% air in an incubator (Thermo-forma, Model No. 371) with controlled humidity at 37°C. Cell preparations with viability more than 95% were used for the experiments. The cells were seeded at a density of 1.0×10^4^ cells/well (counted on hemocytometer) in 0.1% collagen pre-coated 96 well plates, and 7.5×10^5^ cells in 75 cm^3^ flask were used for experiments after being cultured for 24 h. Equal concentration of mannitol was used as osmolar control. Two different treatment regimes were used. In co-treatment cells were exposed to BC-HDD NPs/BBR and high glucose (40 mM) simultaneously for 1.5 h. In post treatment after the high glucose exposure for 1.5 h, media was changed and BC-HDD NPs/BBR was added for 30 min before cells were harvested. Descriptive illustration of same is given below. Experiment groups were named as CNB, CB, PNB and PB which represent co-treatment of BC-HDD NPs, co-treatment of Bulk Berberine chloride, post-BC-HDD NPs and post- Bulk Berberine chloride respectively.

### Cell Viability Assay

Mitochondrial metabolic activity in hepatocytes, following the treatment schedule, was determined by MTT assay as described by Mosmann et al. [22]. The data is expressed as percentage of viability in control cells.

### Uptake of BC and BC-HDD NPs by the Cells

To assess the uptake of berberine (BBR) within the cells, the fluorescence of berberine was detected by flow cytometry [23]. After treatment, the cells were harvested in cold PBS and the fluorescence was measured through the FL1-H filter of flow cytometer (BD-LSR). The data was analyzed in the Cell Quest software supplied with the instrument. Each determination is based on mean fluorescence intensity of 10,000 events.

### Antioxidant Status

#### Superoxide dismutase (SOD) activity

SOD activity was measured according to Kakkar et al [24]. The assay is based on the spectrophotometric assessment of the inhibition of nitro blue tetrazolium-NADH and phenazine methosulphate (PMS) mediated formazan formation. Absorbance was measured at 560 nm. Fifty percent inhibition of formazan formation under assay condition in 1 min is taken as one unit of enzyme activity/minute.

#### Assessment of Nitric oxide (NO)

Accumulation of nitrite in the culture medium, the end product of NO metabolism, was determined using Greiss reagent. In brief, 100 µl of the cell supernatant (1×10^4^ cells/100 µl) was incubated with 100 µl of Greiss reagent (1% sulphanilamide, 0.1% naphthylethylenediamine dihydrochloride and 2.5% H_3_PO_4_) for 30 min at 37°C and the absorbance was recorded at 542 nm. A range of concentration of sodium nitrite was used to generate the standard curve [25].

#### Determination of lipid peroxidation

Malondialdehyde (MDA), the end product of membrane lipid peroxidation, was measured according to Walin et al [26]. To a 10 µl cell lysate, 70 µl of double distilled water, 50 µl of 50 mM phosphate buffer, 10 µl of 1 mM butylated hydroxy toluene (BHT) and 75 µl of 1.3% thiobarbituric acid (TBA) were added. The lipids were precipitated with 50 µl of 50% trichloroacetic acid (TCA). The reaction mixture was then incubated for 40 min at 60°C and then kept on ice for 15 min. The reaction was stopped by adding 10 µl of 20% sodium dodecyl sulphate. The absorbance was taken at 530 nm and 600 nm [26]. 1,1,3,3′Tetraethoxypropane was used as standard.

#### GSH content

Glutathione (GSH) is a major natural intracellular antioxidant, which maintains redox homeostasis. Total GSH was measured spectro-fluorometrically as described earlier [27] using chloromethylfluorescein diacetate (CMF-DA). The treated cells were incubated with the fluorescent dye (5 µg/ml) for 30 min in dark at 37°C and then read at Excitation/Emission wavelength of 485/530 nm on a spectrofluorometer (Synergy HT, Biotek, USA).

### Measurement of ROS Production

ROS generation in hepatocytes was measured by the method of Mohammad et al [28] using the cell permeable fluorescent dye 2′,7-dichlorofluoresceindiacetate (DCFH-DA). The intracellular esterases cleave the diacetate from DCFH-DA to generate DCFH, which is oxidized to DCF (Dichlorofluorescein) by the oxidants and its fluorescence is a measure of ROS production in the cell. The fluorescence intensity was measured on a BD flow cytometer at excitation and emission wavelengths of 485 nm and 530 nm, respectively.

### Mitochondrial Membrane Potential (ΔΨm)

Rhodamine123 fluorescent dye was used to assess the effect of the test substances on mitochondrial membrane potential. Cells were incubated with 10 µl of Rhodamine 123 dye (10 µg/ml in Milli Q water) for 30 min at 37°C. The cells were washed three times and fluorescence was read at 485 nm and 530 nm, excitation and emission wavelengths, respectively as described earlier [29].

### Immunoblot Analysis

Cytosolic and mitochondrial fractions were prepared as described by Tripathi et al [27]. The protein was quantified using Lowry’s method [30]. Samples were incubated with loading dye for 5 min at 96°C and immediately kept on ice. Protein samples (60 µg) from cytosolic fraction were separated by electrophoresis on a 12% SDS– polyacrylamide gel and electro-blotted on a PVDF membrane (HybondTM–P Amersham Biosciences, UK limited, NA). After blocking non-specific sites with 1×blocking buffer, washing was performed using Tris Buffer Saline (TBS) containing 0.1% Tween 20. The membrane was then incubated for 1 h with goat polyclonal IgG antibodies of Bax, Bcl-2 and rabbit polyclonal IgG antibodies of Caspase-3 and Caspase-9 in 1∶500 dilutions and β-Actin (Sigma-aldrich MO, USA) in 1∶1000 dilution. Immunostaining was carried out by incubating the membrane with Horse-Radish Peroxidase-conjugated Rabbit anti-goat IgG, or goat anti-rabbit IgG or goat anti-mouse IgG secondary antibodies (1∶1000) (Santacruz Biotech Inc.) for 1 h at 37°C. Visualization of the immune- positive bands was done using Immobilon™ Western Chemiluminescent HRP substrate kit (Millipore, Corporation, MA, USA). PageRuler™ Prestained Protein Ladder (5 µl), (SM-0671 from Fermentas, EU) was used to determine molecular weight of the protein bands. NIH software Image J version 1.41 (USA) was used to do the densitometric analysis. Band areas were calculated by densitometric scanning and result expressed as arbitrary units for each experimental band.

### Annexin V-FITC Binding Assay

The externalization of phosphatidylserine to the outer surface of cell membrane was detected by fluorescein isothiocyanate (FITC) tagged Annexin V stain, using Annexin V-FITC Apoptosis detection kit (APOAF; Sigma, St. Louis, MO, USA) essentially following the manufacturer’s instructions. To the hepatocytes, Annexin V fluorescein isothiocyanate (FITC) was added and after an incubation of 15 min, propidium iodide (PI) was added to distinguish the necrotic cells based on the following characteristics. (a) Viable hepatocytes, negative for both Annexin V and PI, (b) early apoptotic hepatocytes positive for Annexin V and negative for PI, (c) late apoptotic cells stained positive for both Annexin V and PI, (d) necrotic cells, only PI positive stain. Acquisition of stained cells was done on a flow cytometer (BD-LSR) and analysis performed using Cell Quest software. Each determination is based on the acquisition of 10,000 events [27].

### Apoptotic DNA Content

To determine the population of apoptotic cells, analysis was done using propidium iodide (PI) staining [31]. Hepatocytes were collected and centrifuged at 800 g for 5 min, washed once with cold PBS (0.5 ml) and fixed in 70% chilled ethanol. Fixative was decanted and to the fixed cells 0.1% Triton X-100 was added. Cells were again washed with PBS and resuspended in PBS containing 50 mg/ml PI and 1 mg/ml RNase A for 30 min in the dark at 4°C. Labelled nuclei were subjected to flow-cytometric analysis and then gated on light scatter to remove debris. The percentage of nuclei with sub-G1 content was considered apoptotic cells. PI fluorescence was measured through a FL-2 filter (585 nm).

### Hoechst Staining

Change in the nuclear morphology was observed using bisbenzimide (Hoechst 33258) fluorochrome that binds with DNA. Primary hepatocytes were fixed in ice cold paraformaldehyde (4% in PBS) for 30 min at 37°C followed by permeabilization by cold methanol for 15 min. After washing with PBS, cells were stained with Hoechst 33258 (5 µg/ml) for 10 min. Cells were then observed under a fluorescence microscope (Nikon Eclipse Ti, Model-TI-DH, Japan).

### Statistical Analysis

Data are expressed as mean ± SE. Data were analyzed on a SPSS software version 14.0 using one-way ANOVA and student’s t-test. *P<0.05, **P<0.01, ***P<0.001 were used as the criterion for significance.

## Results

### Preparation and Characterisation of NPs

Biodegradable polymers have been extensively evaluated for the controlled release of pharmacologically active substances. Micro- and nanoparticle formulations of these polymers have been reported in the literature [12]. However, difficulties have been faced to entrap the lipophilic substances inside the hydrophilic polymeric nanoparticles due to low affinity between the lipophilic drug and the polymeric matrix. Therefore, in the present study, dextran has been modified with the attachment of hexadecyl chains via ether linkages to make it amphiphilic. This formulated O-hexadecyl-dextran was allowed to self-assemble in aqueous media entrapping berberine into the hydrophobic pockets producing nanoparticles. The nano-formulation was evaluated for its cytoprotective efficacy in high glucose stressed primary hepatocytes. The drug was entrapped with entrapment efficiency of ∼17%. Drug encapsulation experiment was performed several times by varying the ratio of drug: polymer. The highest encapsulation efficiency was obtained at drug: polymer ratio of 1∶6 (24.28±1.18 mg drug/gram of nanoparticles; table S1). The resulting BC-HDD NPs were characterized for their particle size, zeta potential and kinetic stability. The particle size, determined by dynamic light scattering, revealed the formation of uniformly mono-dispersed NPs with mean particle size being 238±18 nm (PDI = 0.15).

### Size, Surface Morphology and Release Pattern of NPs

**Figure 1:**
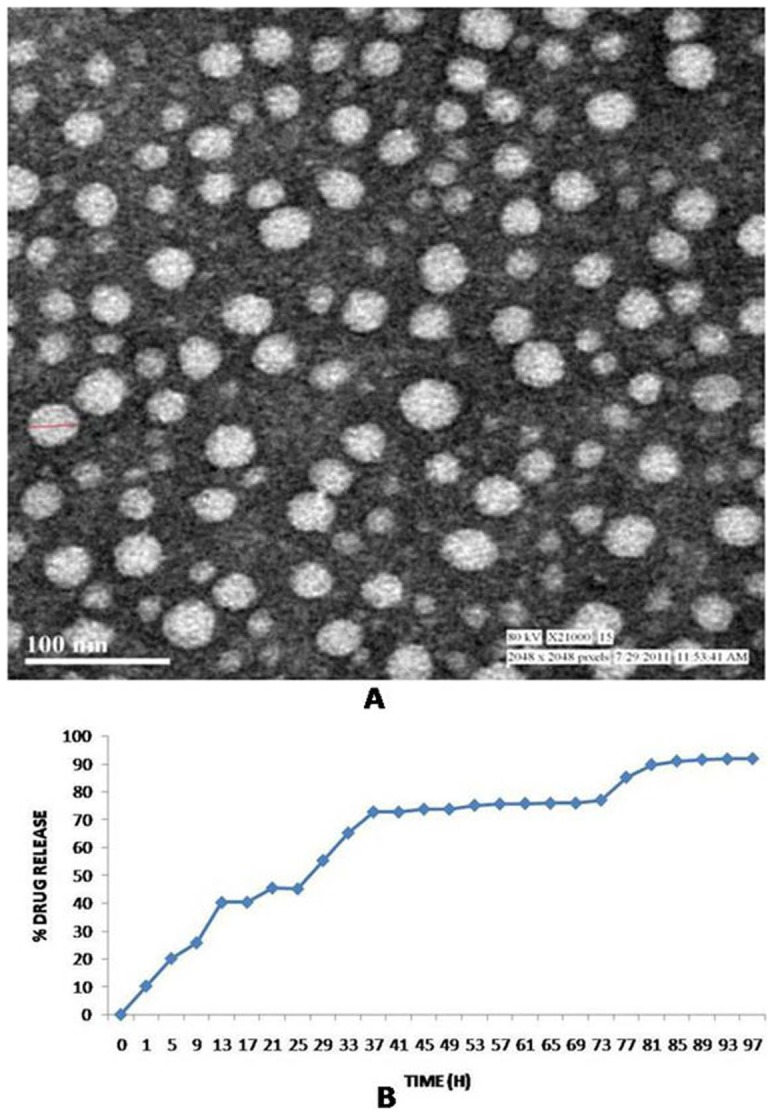
Physiochemical characterization of berberine chloride loaded HDD NPs. (a) Transmission electron microscopy of berberine chloride loaded HDD NPs(BC-HDD NPs) depicting the spherical nature of the nanoparticles having more or less uniform size. Scale bar is 100 nm. (b) *In vitro* drug release following standard dialysis method exhibited by berberine chloride dextran nanoparticles in PBS over a period of 96 hrs. Values are represented as ± SD.

The surface morphology of the BC-HDD NPs was determined by TEM. Figure 1a illustrates a TEM scan showing the formation of spherical nanoparticles having more or less uniform size distribution with particles size in the range of 30–40 nm where as the DLS revealed mono-dispersed NPs with mean particle size being 238±18 nm. The modified hydrophilic polymer, dextran, used in the study has a tendency to bind water. This may have resulted in the formation of particles of larger size, while estimated size by TEM (dehydrated) was found to be 30–40 nm. Similar variation in estimated size was also observed in an earlier study by the group [32]. In vitro release pattern of berberine chloride from the BC-HDD NPs is shown in Figure 1b following the standard dialysis method, which revealed that ∼40% entrapped BC is released within the first 12 h, while in the next 48 h, ∼75% BC was released in a controlled fashion indicating availability of the drug for a longer duration.

### Effective dose of Berberine Chloride and BC-HDD NPs

**Figure 2:**
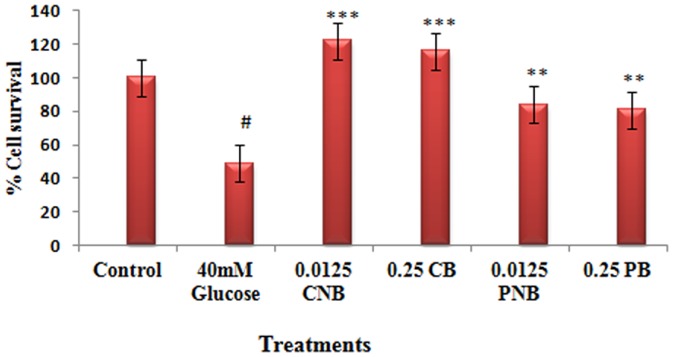
Effective *dose of* berberine chloride and BC-HDD NPs. Cell viability was assessed by MTT Reduction Assay. Results are shown as mean ± S.E. from three independent experiments. Significant difference compared with control values *P<0.05, **P<0.01 and ***P<0.001. Where CNB: Co-BC-HDD NPs; CB: Co-BBR; PNB: Post-BC-HDD NPs and PB: Post-BBR.

Figure 2 shows the results of MTT assay performed to assess the effect of BBR and BC-HDD NPs on glucose stressed primary rat hepatocytes. The cells treated with 40 mM glucose showed a 50% decrease in cell viability. Cells were incubated with increasing concentrations of BBR (0.125 µg to 2.0 µg) and 10 fold lower concentrations of BC-HDD NPs (0.0125 to 0.2 µg) to assess their effect on cell viability (data not shown). In case of BBR and BC-HDD NPs co-treatment, highest cell viability was found to be 110% (P<0.001), at a concentration of 0.25 µg for BBR and 0.0125 µg for BC-HDD NPs, respectively. However, in the case of post-treatment, the cell viability as compared to glucose treated cells was 81% and 84% (P<0.01) both for 0.25 µg for BBR and 0.0125 µg for BC-HDD NPs. It is evident from the data that BC-HDD NPs exhibited similar response in cell viability at twenty-times lesser concentration to that of native BBR.

### Uptake of Berberine Chloride and BC-HDD NPs by the Cells as Assessed by Flow Cytometry

**Figure 3:**
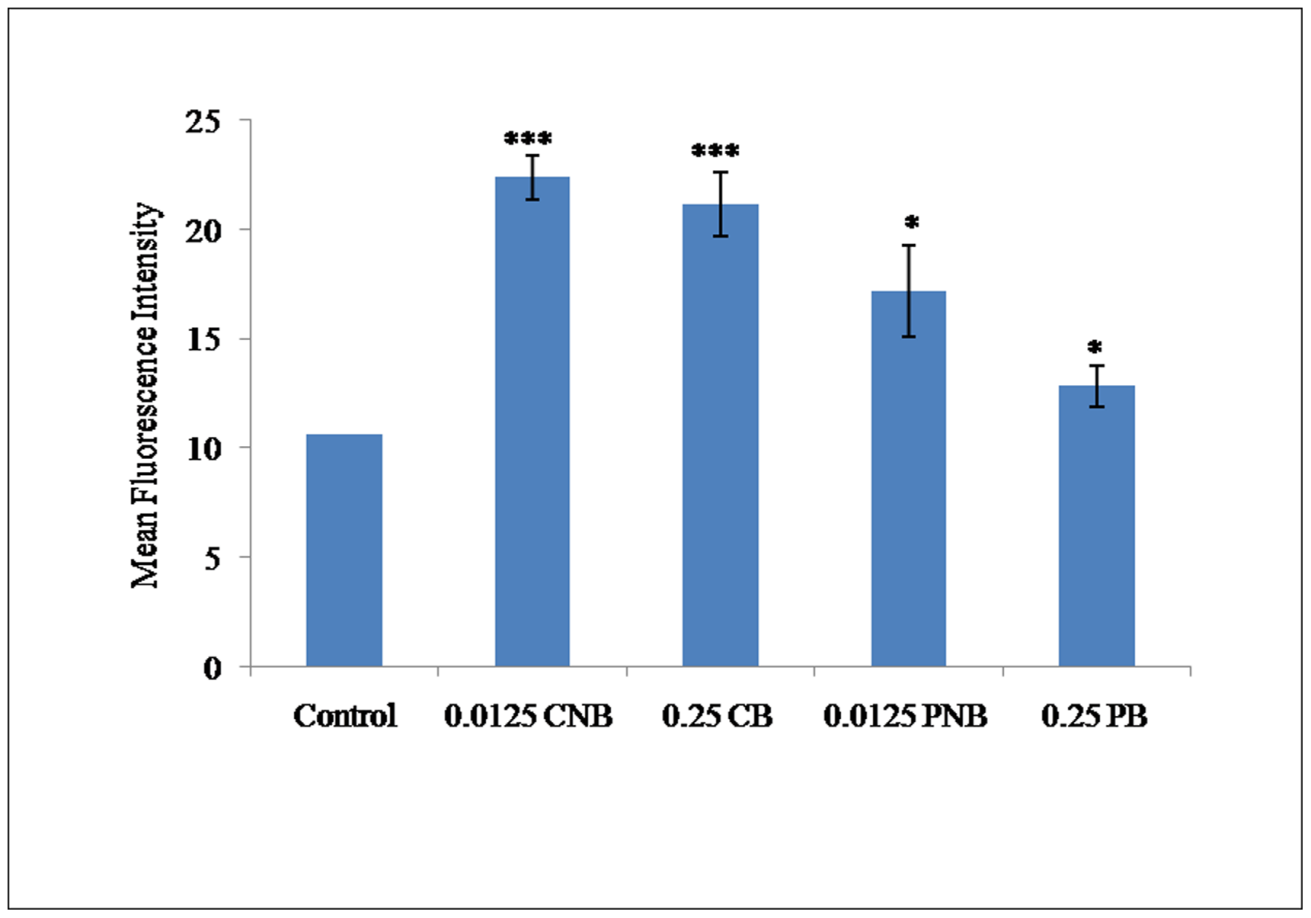
Flow cytometric analysis of uptake of BBR and BC-HDD NPs by the primary rat hepatocytes. The cellular uptake in case of co-treatment was observed after 90 min of treatment whereas in case of post-treatment the uptake was observed after 30 min of BBR and BC-HDD treatment (as per the treatment schedule in the study). Results are shown as mean ± S.E. from three independent experiments. Significant difference compared with control values *P<0.05, and ***P<0.001. Where CNB: Co-BC-HDD NPs; CB: Co-BBR; PNB: Post-BC-HDD NPs, PB: Post-BBR.

Berberine is a fluorescent molecule whose uptake in the cells can be studied by flow cytometry [23]. Therefore, the cells were co-treated and post-treated with 0.25 µg of BBR and 0.0125 µg of BC-HDD NPs (Fig. 3). The cells were subjected to flow cytometry to analyse the uptake of both forms of Berberine within the cells at the selected doses. In the case of co-treatment of cells undergoing hyperglycaemic stress, it was found that the fluorescence intensity due to of BBR was 21.13 (p<0.001) and BC-HDD NPs was 22.35 (p<0.001) indicating that nanotization increased the availability of berberine to the extent that even at 20 fold lower concentration its absorbance within the cell is same as bulk berberine. In the case of post-treatment the absorbance for 0.25 µg BBR was 12.80 (p<0.05) and for 0.0125 µg BC-HDD NPs it was 17.16 (p<0.01) again indicating that nanotization increases bioavailability.

### Antioxidant Status

#### SOD activity

**Figure 4:**
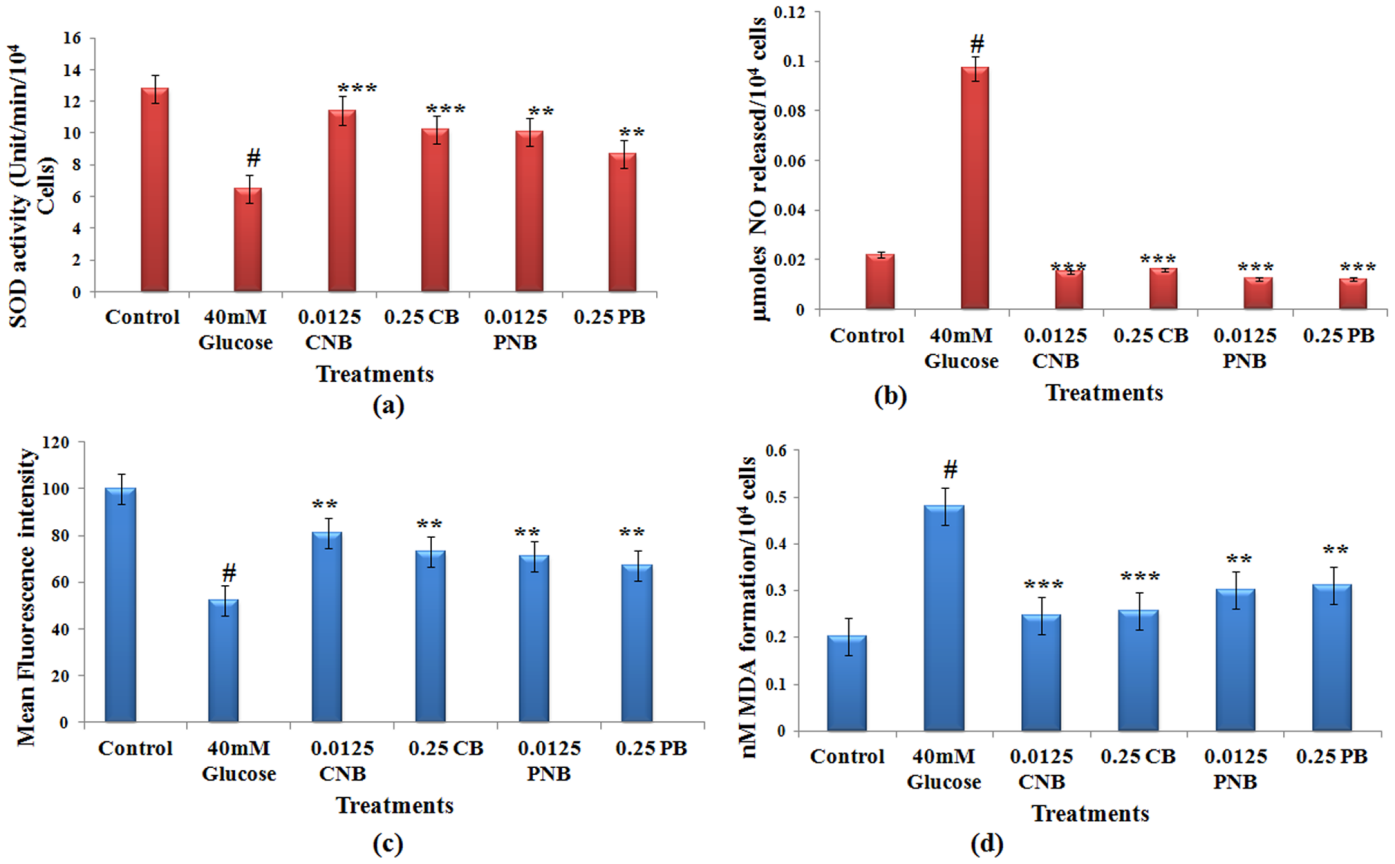
Effect of BBR and BC-HDD NPs on antioxidant status of high glucose stressed hepatocytes. Effect of BBR and BC-HDD NPs on high glucose induced change in SOD activity. (**B**) Effect of BBR and BC-HDD NPs treatment on NO quenching capacity in high glucose stressed hepatocytes. (**C**) Effect of BBR and BC-HDD NPs on GSH content of high glucose stressed hepatocytes. (**D**) Effect on MDA formation on high glucose stressed hepatocytes. Results are shown as mean ± S.E. # denotes significant difference compared with control values *P<0.05, **P<0.01 and ***P<0.001 denotes significant difference compared with 40 mM glucose treated cells. Results are representative of three separate experiments. The S.D. was below ±5% in all cases. Where CNB: Co-BC-HDD NPs; CB:Co-BBR; PNB: Post-BC-HDD NPs and PB: Post-BBR.

Hepatocytes, under hyperglycaemic stress showed significant decrease in SOD activity i.e. 6.0 U/min/1×10^4^ cells as compared to the untreated cells with the SOD activity of 13.0 U/min/1×10^4^ cells. On co- and post-treatment with both forms, this decline was prevented with a significant restoration of SOD activity. In co-treatment the activity was 10.23 U/min/1×10^4^ cells (P<0.01) for 0.25 µg BBR and 11.41 U/min/1×10^4^ cells (P<0.01) for 0.0125 µg BC-HDD NPs respectively (Fig. 4a). During post treatment the SOD activity was found to be 8.68 U/min/1×10^4^ cells in BBR treated cells and 10.66 U/min/1×10^4^ cells in BC-HDD NPs treated cells. It is evident that a 20 fold lower dose of BC-HDD NPs could elicit better response in maintaining antioxidant status.

#### NO release

Figure 4 b shows accumulation of nitrite as an index of NO release, in the culture medium. BBR and BC-HDD NPs were effective in decreasing NO release. In glucose treated cells the release was increased by 4.4 fold (P<0.001), which was effectively decreased to 0.7 and 0.6 fold in case of co and post BC-HDD NPs treatment. Whereas, effect in the case of co- and post-treatment of BBR was found to be 0.8 and 0.6 fold respectively (Fig. 4b).

#### Lipid peroxidation

Level of lipid peroxidation during oxidative stress is an indicator of excessive cellular damage. Hepatocytes exposed to high glucose exhibited significant increase in the level of MDA formation (0.48 nM MDA/10^4^ cells; P<0.001) as compared to 0.20 nM MDA formation/10^4^ cells in control which was prevented by both BBR and BC-HDD NPs. In case of co-treatment the level was 0.26 nM MDA/10^4^ cells in BBR treated cells and 0.25 nM MDA/10^4^ cells (P<0.01) in case of BC-HDD NPs. However, in post treatment, it was 0.31 nM MDA/10^4^ cells in case of BBR and 0.30 nM MDA/10^4^ cells for BC-HDD NPs (Fig. 4c) eliciting comparable response at 20 fold lower concentration of BC-HDD NPs.

#### GSH content

The level of GSH was determined by flow cytometry using CMF fluorescent probe. GSH content decreased by 48% in glucose stressed cells, which was restored by 21% during co-treatment by BBR and 29% by BC-HDD NPs. Response of BBR and BC-HDD NPs in post treatment was similar (18 and 19% respectively), but less effective to that observed with the co-treatment (Fig. 4d).

### ROS Generation during High Glucose Stress and Prevention by BC-HDD NPs

**Figure 5:**
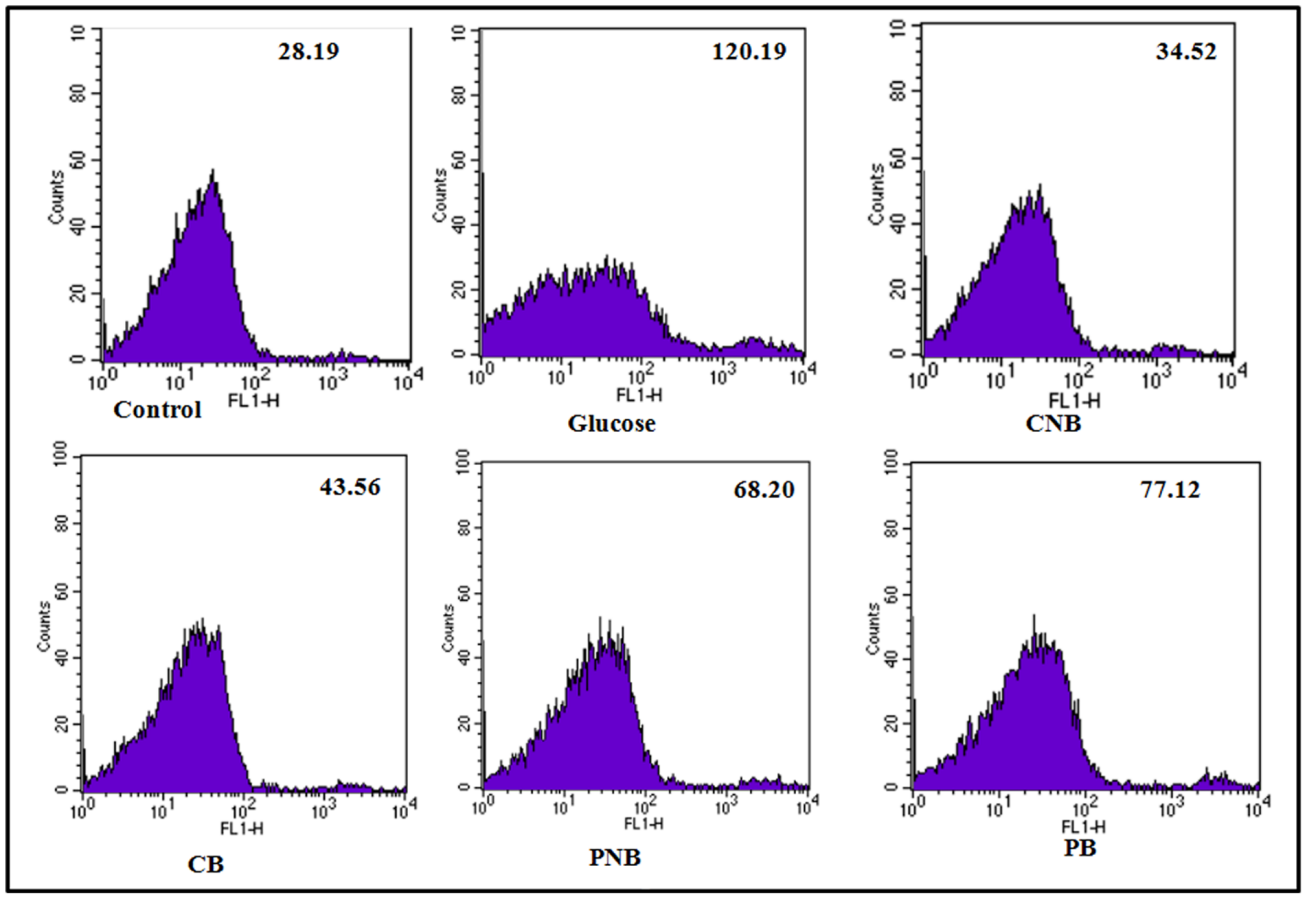
Effect of BBR and BC-HDD NPs on ROS production in high glucose treated hepatocytes. DCFH-DA dye was used to assess the ROS production. Data shown are mean evaluated from three different sets of experiments. The S.D. was below ±5% in all cases. Where CNB: Co-BC-HDD NPs; CB:Co-BBR; PNB: Post-BC-HDD NPs and PB: Post-BBR.

Intracellular ROS generation during different exposure conditions was estimated using DCFH-DA fluoroprobe. High glucose treated cells exhibited ∼4.3 fold increase in ROS generation. Cells co-treated with BBR and BC-HDD NPs, showed a significant (P<0.001) decline in the ROS generation by ∼2.8 and ∼3.5 fold, respectively, as compared to glucose stressed cells. While cells post-treated with BBR and BC-HDD NPs displayed ∼1.6 and 1.8 fold decrease in ROS generation, respectively (Fig. 5). The data indicates significant prevention of ROS generation during co-treatment of BC-HDD NPs.

### BC-HDD NPs Prevent Loss of Mitochondrial Membrane Potential

**Figure 6:**
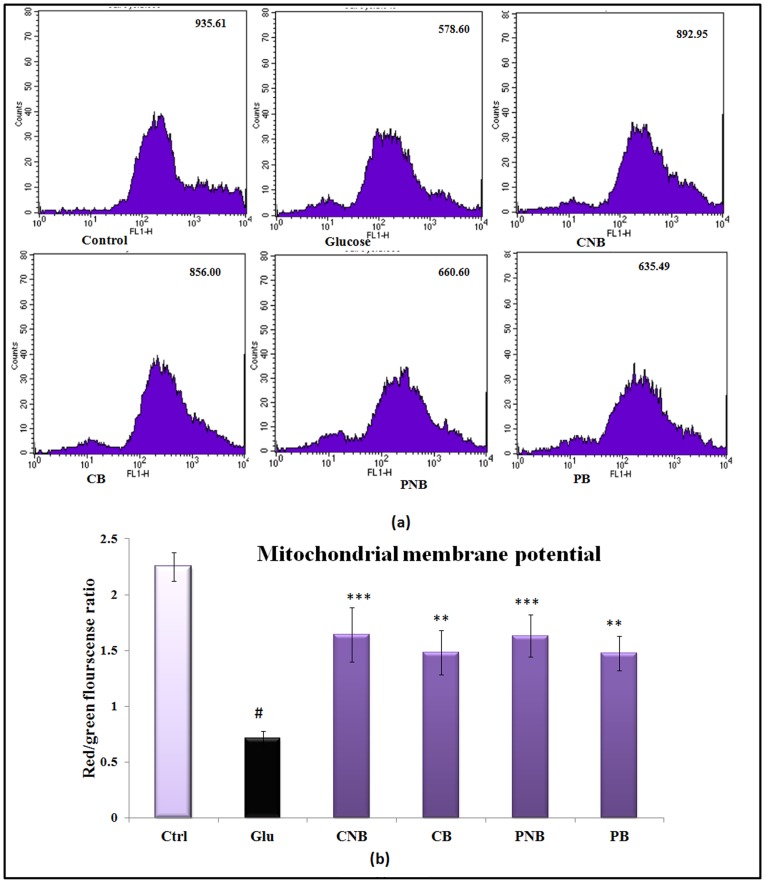
BC-HDD NPs prevent loss of mitochondrial membrane potential. (**A**) Effect of BBR and BC-HDD on mitochondrial polarisation as seen using Rhodamine123. (**B**) Effect on mitochondrial membrane potential as seen using JC-1. Results are shown as mean ± S.E. # denotes significant difference compared with control values and *P<0.05, **P<0.01 and ***P<0.001 denotes significant difference compared with 40 mM glucose. Where CNB: Co-BC-HDD NPs; CB:Co-BBR; PNB: Post-BC-HDD NPs and PB: Post-BBR.

The accumulation of fluorescent dye Rhodamine 123 was taken as an indicator of mitochondrial membrane potential. Significant decrease in MMP, i.e. 38.2% (P<0.01), was observed in high glucose stressed cells as compared to control cells. BBR and BC-HDD NPs prevented depolarization of mitochondria and showed only 8.5% (P<0.01) and 4.6% (P<0.01) decrease in mean fluorescent intensity, respectively, during co-treatment. In post-treatment, restoration of MMP was 9.8 (P<0.05) and 14% (P<0.05) for BBR and BC-HDD NPs, respectively (Fig. 6a). In addition to the results observed by flow-cytometry, an increase of monomeric JC-1 molecules (green fluorescence) due to a decrease of ΔΨm occurred in glucose stressed hepatocytes. Red to green fluorescence ratio decreased ∼3 fold (P<0.001) in stressed hepatocytes as the mitochondria became progressively depolarized. In BBR and BC-HDD NPs treated cells, ratio of polarized mitochondria was increased by ∼2.1 and 2.3 fold, respectively (P<0.001) in comparison to glucose stressed hepatocytes, maximum being in co-treated cells with BC-HDD NPs (Fig. 6b).

### Level of Apoptotic and Antiapoptotic Proteins

**Figure 7:**
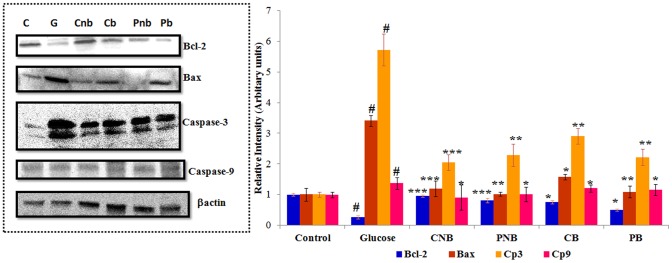
Level of apoptotic and antiapoptotic proteins. Immunoblot showing expression of Bcl2, Bax, caspase-3 and caspase-9 in cytosolic fraction. *β*-actin serving as internal control. Graph shows fold change in expression level. Results are shown as mean ± S.E. # denotes significant difference compared with control values and *P<0.05, **P<0.01 and ***P<0.001 denotes significant difference compared with 40 mM glucose. Where CNB: Co-BC-HDD NPs; CB: Co-BBR; PNB: Post-BC-HDD NPs and PB: Post-BBR.

Bcl-2 is an anti-apoptotic protein, which helps the cells in preventing apoptosis. In high glucose treated cells, expression level of Bcl-2 protein was decreased by ∼74% (P<0.001), which was ameliorated by the treatment of BBR, wherein co-treatment decrease was found to be ∼24% and in post-treatment 50%. On the other hand, for BC-HDD NPs, the co-treatment showed a decrease of 4% (P<0.001) only and 19% in post-treatment. However, expression of apoptotic protein bax was found to be up-regulated by ∼3.4 fold (P<0.01) under hyperglycaemic stress. This expression was downregulated by BC-HDD NPs (∼1.2 fold in co-treatment and ∼1.6 fold in post-treatment) and BBR (1 fold in co-treatment and 1.1 fold in post treatment) respectively. Similarly, expression of activated caspase-3 and caspase-9 increased in glucose stressed cells by ∼5.7 (P<0.001) and 1.4 fold respectively. This activation of caspases was prevented significantly on treatment with BBR i.e., ∼2.3 fold in co-treatment and 2.2 (P<0.01) fold in post-treatment for caspase-3; ∼1.5 fold in co-treatment and ∼1.2 (P<0.05) fold in post-treatment for caspase-9. BC-HDD NPs treatment also prevented activation of caspases i.e it was ∼2.1 fold in co-treatment and ∼2.9 fold in post-treatment for caspase-3; while for caspases-9 its level was found to be equivalent to that in control in both the treatments (Fig. 7). The data again supports our earlier findings that BC-HDD NPs at 20 fold lower concentration elicit similar protection to stressed cells.

### Annexin V-FITC Binding Assay

**Figure 8:**
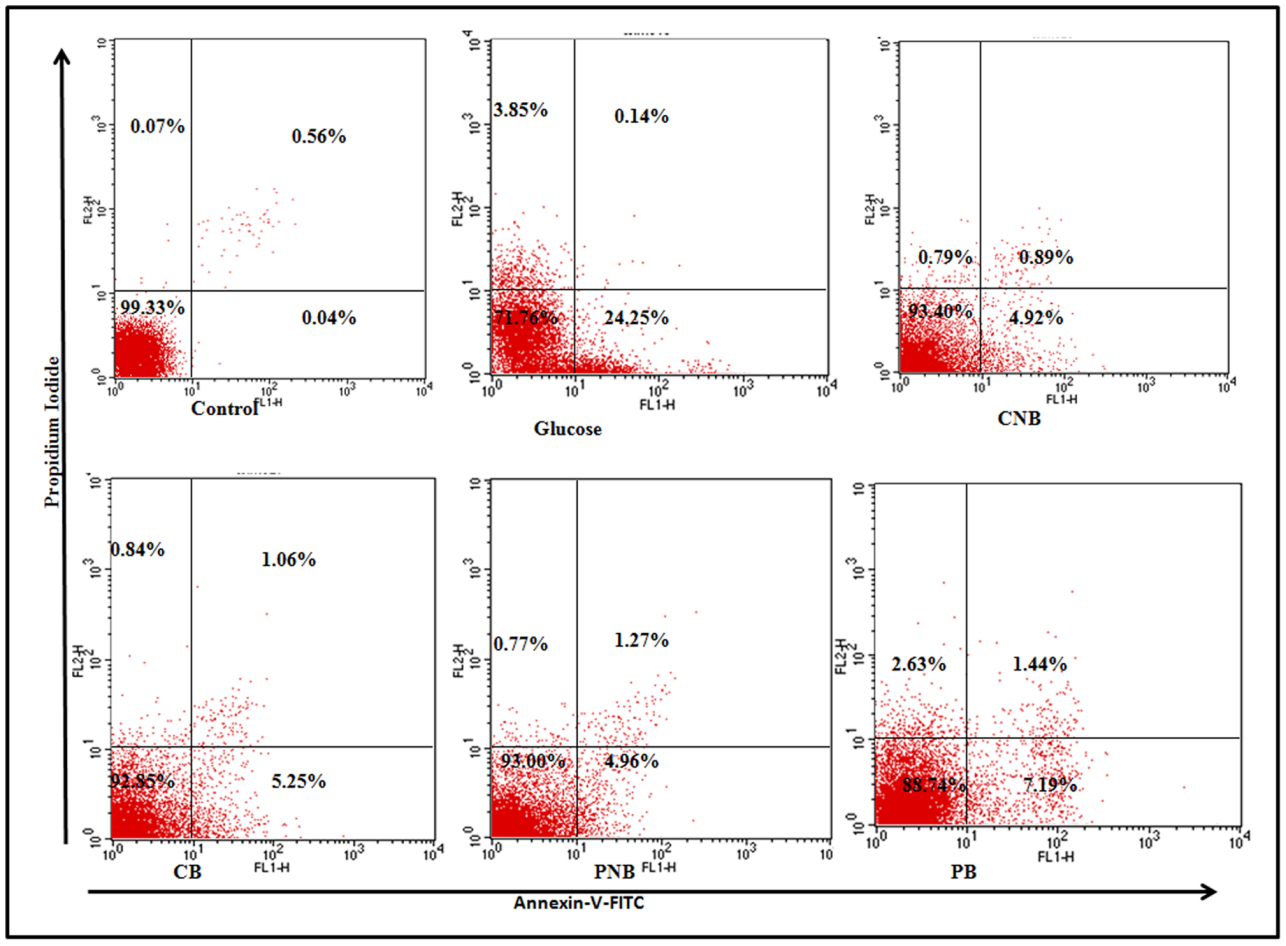
Modulation in hyperglycemia induced apoptosis by BBR and BC-HDD. Effect of BBR and BC-HDD NPs on hyperglycemia induced apoptosis in hepatocytes as assessed by Annexin-PI staining. The S.D. was below ±5% in all cases. Results are representative of three separate experiments. Where CNB: Co-BC-HDD NPs; CB: Co-BBR; PNB: Post-BC-HDD NPs and PB: Post-BBR.

Apoptosis in glucose stressed primary rat hepatocytes was assessed by annexin V-FITC binding assay. In control cells, ∼99.3% of the cell population was found to be viable. In the cells treated with glucose, ∼71.8% cells were viable, 24.3% in early apoptosis, 0.1% in late apoptosis, whereas, ∼3.9% cells were undergoing necrosis. In co-treatment, BC-HDD NPs showed only 4.9% cells in early apoptosis, 0.9% in late apoptosis, 0.8% cells undergoing necrosis and remaining 93.4% cells were found to be viable confirming highly significant prevention of cytotoxicity due to high glucose stress. BBR also showed similar response i.e. 5.3% cells were in early apoptosis, 1.1% in late apoptosis, 0.8% cells undergoing necrosis and remaining 92.9% cells were found to be viable. In post-treatment with BC-HDD NPs, 5% cells were found to be in early apoptosis, and 93.0% cells were found to be viable, whereas, post-treatment with BBR showed 7.2% cells in early apoptosis, and 88.7% cells were found to be viable (Fig. 8).

### Apoptotic DNA Content

**Figure 9:**
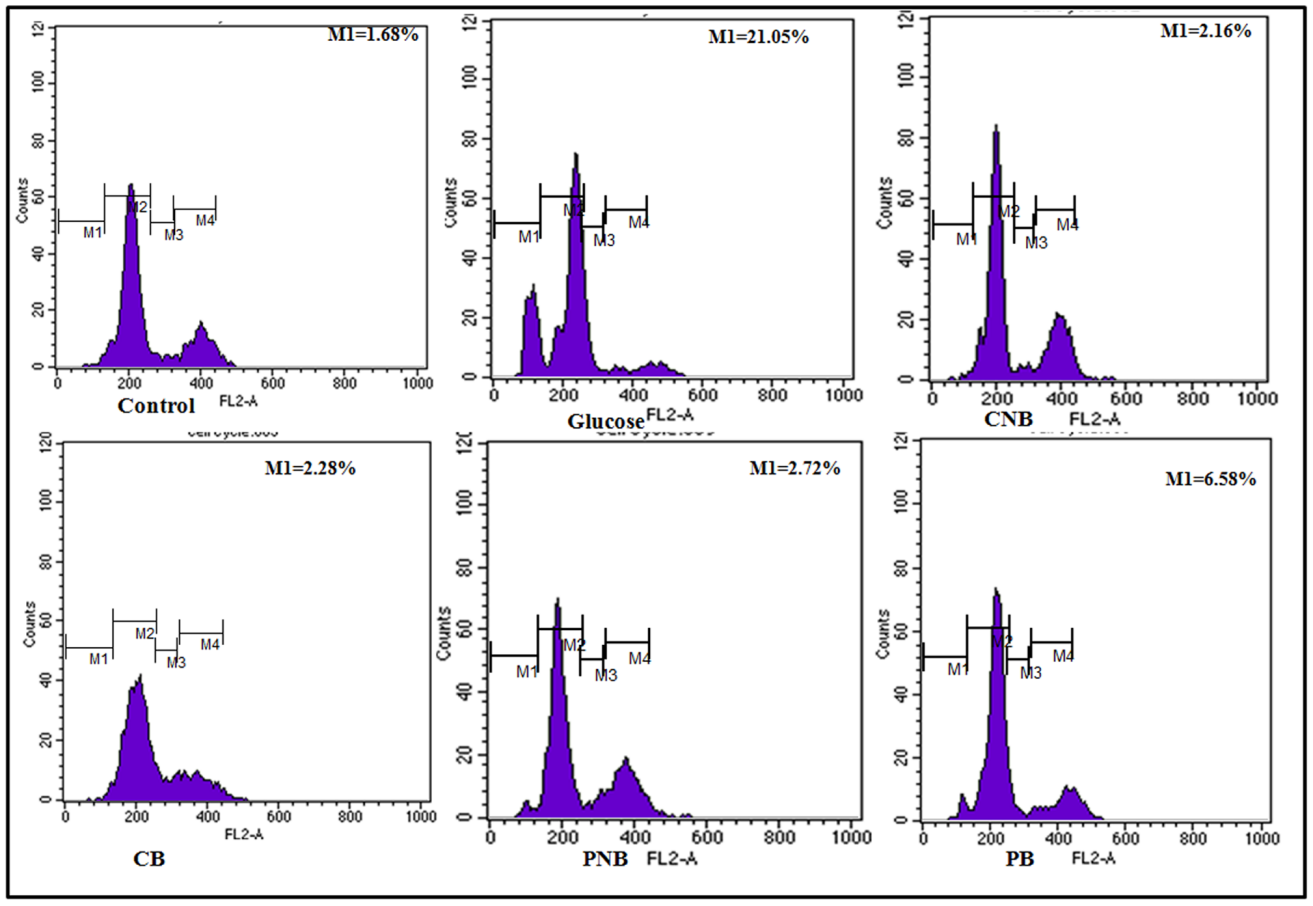
Effect of BBR and BC-HDD on hyperglycemia induced changes in cell cycle. Effect of BBR and BC-HDD on cells treated with glucose. Results expressed as the % of sub-G1 population. The propidium iodide fluorescence was measured using flow cytometer with FL-2 filter. S.D. was below ±5% in all the cases. Results are representative of three separate experiments. Where CNB: Co-BC-HDD NPs; CB:Co-BBR; PNB: Post-BC-HDD NPs and PB: Post-BBR.

Cell cycle analysis with cellular DNA content was performed by flow cytometry. In cell cycle studies, the number of sub-diploid cells after glucose treatment was found to be 21.05% with respect to that of 1.45% in control. Cells co-treated with BBR and BC-HDD NPs decreased the apoptotic DNA content to 2.3% and 2.2%, respectively. Post treatment with these formulations reduced the apoptotic DNA content to 6.6% and 2.7%, respectively (Fig. 9). Here, BC-HDD NPs were found to be effective during post-treatment also.

**Figure 10:**
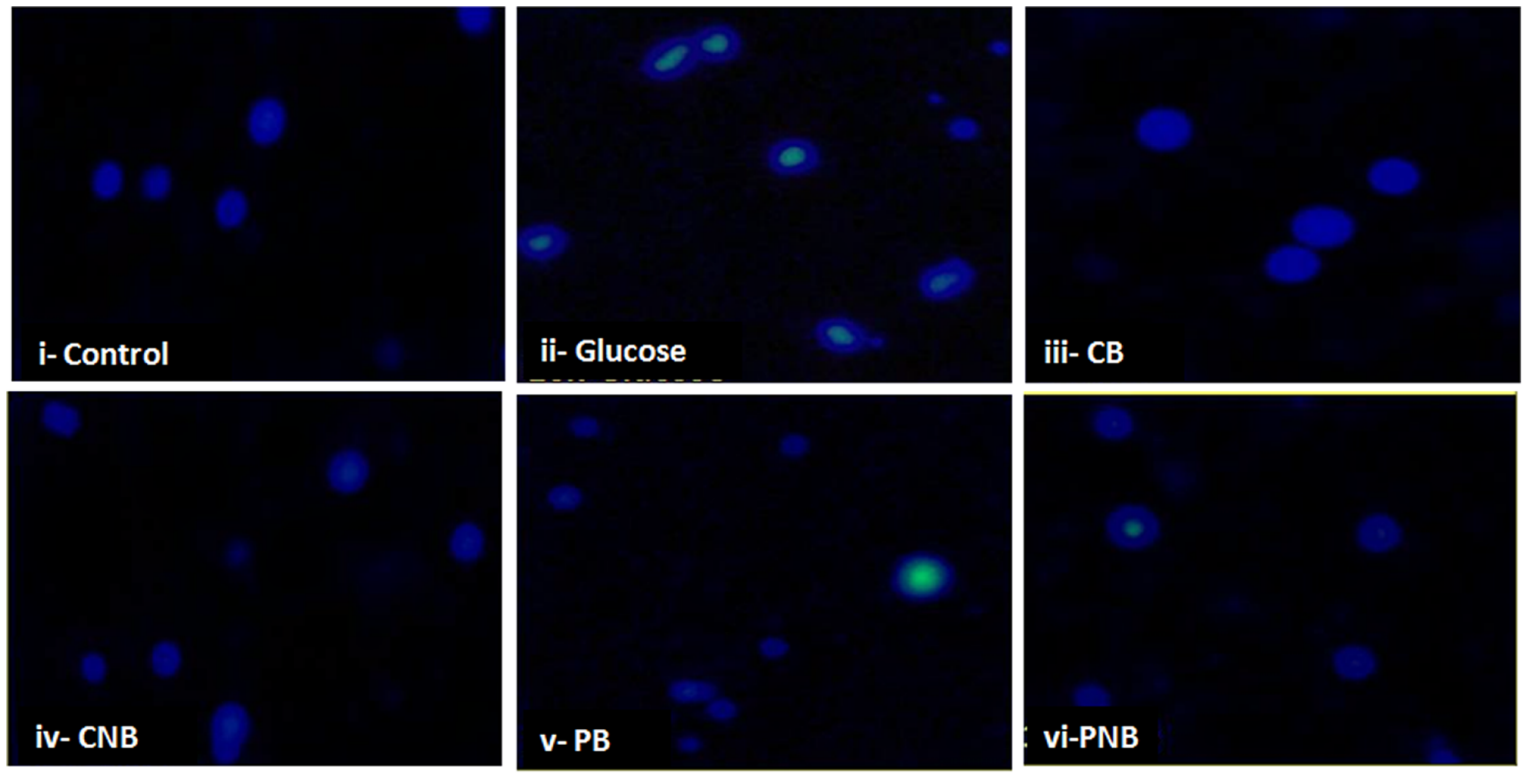
Chromatin condensation occurred during hyperglycemia and protection by BBR and BC-HDD. Rat hepatocytes were stained with Hoechst 33258 and visualized at 10×magnification to observe chromatin condensation. (i) Control, (ii) 40 mM glucose, (iii) CB: Co-BBR, (iv) CNB: Co-BC-HDD NPs (v) PB: Post-BBR (vi) PNB: Post-BC-HDD NPs.

Fluorescence microscopy of fixed cells stained with Hoechst 33258 was used to enumerate cells with chromatin condensation typical of apoptosis (Fig. 10). The cells that were treated with glucose showed increased fluorescence intensity as well as the nuclear chromatin condensation. There was a marked increase in the number of apoptotic cells containing condensed and irregular aggregation of nuclear chromatin, whereas cells treated with BBR and BC-HDD NPs showed no change in nuclear morphology.

## Discussion

The fate of a drug after its administration depends primarily on the physicochemical properties of the drug and on its chemical structure. Therefore, physico-chemical properties of NPs like particle size, zeta potential, colloidal stability, etc, become essential. The widely used approaches are grafting of polyethylene glycol (PEG) [33] or the use of polysaccharides such as dextran [14] as the carrier surface. Durand et al [20] showed that hydrophobically modified dextrans could be used as effective stabilizers. We grafted aliphatic hexadecyl chain to dextran (O-hexadecyl-dextran) via ether linkages in the presence of a base and subsequently encapsulated berberine. It was speculated that physical interactions would take place between the hydrophobic chains of HDD and berberine resulting in the formation of self-assembled NPs (BC-encapsulated HDD) in aqueous media. Subsequent to mixing of BC and HDD NPs in aqueous medium, BC-HDD NPs were obtained with encapsulation of 28.3 mg berberine/g of nanoparticles. Since, particle size can directly affect the physical stability, cellular uptake, biodistribution and drug release from the NPs, BC-HDD NPs were characterized for their size distribution by DLS as well as TEM and further investigated for their anti-diabetic potential *in vitro*. BC-HDD NPs formed were in the size range of 30–40 nm as evident from TEM analysis. *In vitro* release study showed the sustained and continuous release of berberine from the polymer surface.

Hyperglycaemia plays an important role in pathophysiology of diabetes. ROS generation during various metabolic pathways has been attributed as one of the causative factors for development and progression of diabetes [34], [35]. Berberine, an alkaloid, has been reported to have anti-diabetic activity [36], [37]. Moreover, a direct action of berberine on carbohydrate metabolism in the intestine has been suggested in the recent studies [38]–[41]. Short-term clinical trials have also confirmed the anti-diabetic and insulin-sensitizing effect of berberine [38]. The low dosage of BBR has been found to be tolerable to the organism while its higher dosage has been found to cause toxicity along with some gastrointestinal complaints [42], [43]. Due to reported adverse effects of BBR at higher concentrations, it was nanotized and entrapped in O-hexadecyl-dextran (HDD) to obtain BC-HDD NPs. These NPs were found to produce an initial burst of 40% within 12 h, and thereafter, the drug released in a controlled fashion (∼90%) up to 96 h. Our contention is that if the cells take up NPs and release them for a longer period of time, there will be an improved cytoprotection and lesser degree of oxidative stress in glucose stressed hepatocytes. While examining the effect of nanotized formulation on cell viability, the formulation was found to be effective at 20 folds lower concentration and reverted the critical control points of the apoptotic cell death. The flow cytometric analysis also indicated that the fluorescence of BC-HDD NPs taken up by the cells was same as that of 20 fold higher bulk berberine which emphasized on the fact that nanotisation does increases bio-availability. At such a low concentration (0.0125 µg/10^4^cells), BC-HDD NPs were found to prevent lipid peroxidation, NO generation and could prevent decline in SOD activity under severe glucose stress.

Increased ROS generation due to hyperglycemia initiates a cascade of events that may culminate into apoptotic cell death [44]. The oxidative stress alters mitochondrial permeability where Bcl-2 family proteins play a key role in regulating the pore formation. Bcl-2 and Bax couple with each other and prevent permeability alteration. However, when stress arises, this coupling is disrupted leading to formation of Bax dimers, which facilitate pore formation. Bcl-2 is also reported to prevent apoptosis by regulating level of glutathione pool by causing redistribution of GSH, which in turn, then prevents ROS production and GSH depletion thereby preventing apoptosis [45]. The present study, however, clearly demonstrates that during hyperglycaemic stress, an increase in the level of ROS takes place along with the depletion of GSH. This was further accompanied by an increase in the level of Bax and decrease in the level of Bcl-2. This brings out the existence of regulated co-ordination between ROS generation, GSH depletion and Bax/Bcl-2 imbalance.

In our study, glucose stress to the hepatocytes revealed a decrease in the mitochondrial potential, however, when native BBR and BC-HDD NPs were administered, a decrease in the lowering of Δψm was observed. The restoration of MMP was more pronounced in BC-HDD NPs co-treated cells where a 20 fold lower concentration of nanoparticle was found to be effective. This result clearly indicates that BC-HDD NPs’ protective effect is mediated via mitochondrial pathway.

The mitochondria mediated apoptotic pathway further involves formation of a heptameric protein complex, apoptosome which involves release of apoptotic proteins from mitochondria. This complex then acts on the inactivated pro-caspase 9, which is cleaved into active caspase 9. The activated caspase 9 then acts further downstream on other inactivated executioner pro-caspase 3. This leads to activation of caspase 3, which further causes apoptosis. BC-HDD NPs again show a more profound protective effect on the hepatocytes when administered along with glucose where it decreased the activation of caspase 3 and 9. It lowered the level of caspase 9 to a level comparable to the control while caspase 3 was decreased by 2.8 fold as compared to glucose stress response. These results further consolidate the modulation of intrinsic pathway by BC-HDD NPs.

Co- and post-treatment of native BBR and BC-HDD NPs were found to prevent the glucose stressed cells from undergoing apoptosis as assessed by externalisation of phosphatidylserine. The cell cycle results also corroborate with the previous findings as the arrest of hepatocytes in sub-G1 phase observed in glucose treatment was reversed by the action of native BBR and BC-HDD NPs. Our results indicate that cells undergoing apoptosis due to high glucose stress follow intrinsic pathway where an increase in the ROS generation, depolarisation of mitochondria, increase in Bax/Bcl2 ratio, activation of caspase-3 and 9 leading to cell cycle arrest and chromatin condensation were significant hallmarks. Taken together, BC-HDD NPs were effective at 20 folds lower concentration than that of native BBR in modulating critical control points of intrinsic apoptotic pathway. This enhanced effectiveness can be credited to the enhanced availability of berberine to the cells due to its nanotization.

## Conclusion

The study suggests an improved action of berberine in its nanotized form (BC-HDD NPs) when applied to glucose stressed hepatocytes. A decline in ROS generation, oxidative stress, caspase activation and prevention of depolarisation of mitochondria, was observed in BC-HDD NPs treated cells. This was achieved by using a 20 fold dose advantage (lower dose) which was not only due to its nanotized form but also due to its longer availability inside the cells as achieved by the designed and synthesized BC-HDD NPs. The study further raises wider implications for the use of low concentrations of drugs that are effective yet toxic at higher concentrations to the cells for the treatment of diseases/disorders like diabetes.

## Supporting Information

Table S1 **Entrapment efficiency of the BC-HDD nanoparticles.** The entrapment efficiency is expressed in terms of amount of drug (mg) loaded per gram of nanoparticles. Highest entrapment of Berberine was observed at 1∶6 drug polymer ratio.
(DOCX)
