# Supplementary material for: O-Hexadecyl-Dextran Entrapped Berberine Nanoparticles Abrogate High Glucose Stress Induced Apoptosis in Primary Rat Hepatocytes
Source: PLoS One. 2014 Feb 20;9(2):e89124. doi: 10.1371/journal.pone.0089124 (PMC3930636; doi:10.1371/journal.pone.0089124)
Supplement: Table S1 — Entrapment efficiency of the BC-HDD nanoparticles. The entrapment efficiency is expressed in terms of amount of drug (mg) loaded per gram of nanoparticles. Highest entrapment of Berberine was observed at 1∶6 drug polymer ratio. (DOCX) [file pone.0089124.s001.docx]

**Table S1**: **Entrapment efficiency of the BC-HDD nanoparticles**

| S.No. | Drug : Polymer ratio | Entrapment efficiency (%) | Drug Loading (mg of drug/gram of nano-particles) |
| --- | --- | --- | --- |
| 1. | 1:2 | 2 ± 0.15 | 0.66 ± 0.35 |
| 2. | 1:4 | 7 ± 0.21 | 14.0 ± 1.15 |
| 3. | 1:6 | 17 ± 1.01 | 24.28 ± 1.18 |
| 4. | 1:10 | 12 ± 1.0 | 10.90 ± 1.02 |

The entrapment efficiency is expressed in terms of amount of drug (mg) loaded per gram of nanoparticles.
